# Alpha-Fetoprotein- and CD40Ligand-Expressing Dendritic Cells for Immunotherapy of Hepatocellular Carcinoma

**DOI:** 10.3390/cancers13133375

**Published:** 2021-07-05

**Authors:** Annabelle Vogt, Farsaneh Sadeghlar, Tiyasha H. Ayub, Carlo Schneider, Christian Möhring, Taotao Zhou, Robert Mahn, Alexandra Bartels, Michael Praktiknjo, Miroslaw T. Kornek, Marieta Toma, Ingo G. H. Schmidt-Wolf, Vittorio Branchi, Hanno Matthaei, Jörg C. Kalff, Christian P. Strassburg, Maria A. Gonzalez-Carmona

**Affiliations:** 1 Department of Internal Medicine I, University Hospital of Bonn, 53127 Bonn, Germany; Annabelle.Vogt@web.de (A.V.); farsaneh.sadeghlar@ukbonn.de (F.S.); tiyasha_hosne.ayub@ukbonn.de (T.H.A.); carlo.schneider@gmx.de (C.S.); christianmoehring1@gmx.de (C.M.); Taotao.zhou@ukbonn.de (T.Z.); robert.mahn@ukbonn.de (R.M.); alexandra.bartels@ukbonn.de (A.B.); michael.praktiknjo@ukbonn.de (M.P.); Miroslaw_theodor.kornek@ukbonn.de (M.T.K.); christian.strassburg@ukbonn.de (C.P.S.); 2 Department of Pathology, University Hospital of Bonn, 53127 Bonn, Germany; marieta.toma@ukbonn.de; 3 Department of Integrated Oncology (CIO), University of Bonn, 53127 Bonn, Germany; ingo.schmidt-wolf@ukbonn.de; 4 Department of Visceral Surgery, University Hospital of Bonn, 53127 Bonn, Germany; vittorio.branchi@ukbonn.de (V.B.); hanno.matthaei@ukbonn.de (H.M.); joerg.kalff@ukbonn.de (J.C.K.)

**Keywords:** alpha-fetoprotein, CD40Ligand, dendritic cells, hepatocellular carcinoma, subcutaneous and orthotopic murine HCC

## Abstract

**Simple Summary:**

In first clinical trials, vaccinations against tumor-associated antigens (TAA), such as Alpha-Fetoprotein (AFP) using antigen presenting cells, such as dendritic cells (DC), failed to achieve effective immune responses towards hepatocellular carcinoma (HCC). CD40Ligand is a potent immune checkpoint, which can increase the antitumoral immune response of DC. In this study, a subcutaneous vaccination with DCs, which were transduced with AFP-coding adenoviruses and an intratumoral treatment with DCs, which were transduced with CD40L-coding adenoviruses, induced an antitumoral immune response and led to complete remissions and long-term survival in 62% of mice with established HCC. Combined strategy causes rapid and profound changes in the tumor environment with enhanced Th1-cytokine expression, strong tumor infiltration of cytotoxic T lymphocytes and DC, and higher tumor apoptosis, leading to effective tumor regression of HCC. Thus, intratumoral CD40L co-stimulation represents a promising tool for improving tumor-antigen DC-based immunotherapy of HCC.

**Abstract:**

Dendritic cells (DC) as professional antigen presenting cells are able to prime T-cells against the tumor-associated antigen α-fetoprotein (AFP) for immunotherapy of hepatocellular carcinoma (HCC). However, a strong immunosuppressive tumor environment limits their efficacy in patients. The co-stimulation with CD40Ligand (CD40L) is critical in the maturation of DC and T-cell priming. In this study, the impact of intratumoral (i.t.) CD40L-expressing DC to improve vaccination with murine (m)AFP-transduced DC (Ad-mAFP-DC) was analyzed in subcutaneous (s.c.) and orthotopic murine HCC. Murine DC were adenovirally transduced with Ad-mAFP or Ad-CD40L. Hepa129-mAFP-cells were injected into the right flank or the liver of C3H-mice to induce subcutaneous (s.c.) and orthotopic HCC. For treatments, 10^6^ Ad-mAFP-transduced DC were inoculated s.c. followed by 10^6^ CD40L-expressing DC injected intratumorally (i.t.). S.c. inoculation with Ad-mAFP-transduced DC, as vaccine, induced a delay of tumor-growth of AFP-positive HCC compared to controls. When s.c.-inoculation of Ad-mAFP-DC was combined with i.t.-application of Ad-CD40L-DC synergistic antitumoral effects were observed and complete remissions and long-term survival in 62% of tumor-bearing animals were achieved. Analysis of the tumor environment at different time points revealed that s.c.-vaccination with Ad-mAFP-DC seems to stimulate tumor-specific effector cells, allowing an earlier recruitment of effector T-cells and a Th1 shift within the tumors. After i.t. co-stimulation with Ad-CD40L-DC, production of Th1-cytokines was strongly increased and accompanied by a robust tumor infiltration of mature DC, activated CD4^+^-, CD8^+^-T-cells as well as reduction of regulatory T-cells. Moreover, Ad-CD40L-DC induced tumor cell apoptosis. Intratumoral co-stimulation with CD40L-expressing DC significantly improves vaccination with Ad-mAFP-DC in pre-established HCC in vivo. Combined therapy caused an early and strong Th1-shift in the tumor environment as well as higher tumor apoptosis, leading to synergistic tumor regression of HCC. Thus, CD40L co-stimulation represents a promising tool for improving DC-based immunotherapy of HCC.

## 1. Introduction

Hepatocellular carcinoma (HCC) ranks sixth amongst the most frequent malignancies worldwide and its incidence is increasing [1,2]. Most patients are diagnosed with advanced disease eligible only for palliative procedures. Despite the development of new molecular-targeted therapies, such as sorafenib, lenvatinib, regorafenib, cabozantinib and ramucirumab, as well as immune checkpoint inhibitors, such as nivolumab, pembrolizumab and atezolizumab in combination with bevacizumab, the treatment of HCC is still challenging and its prognosis remains extremely poor [3,4,5,6,7,8].

Dendritic cells (DC), as professional antigen presenting cells, are able to initiate a T-cell-mediated immune response against tumor-associated antigens (TAA) [9]. Alpha-fetoprotein (AFP) is a well-known TAA, which is overexpressed in more than 50% of HCC. Previously, we and others demonstrated the presence of an immune response to AFP in patients with HCC [10,11,12], suggesting that AFP may be a useful target for vaccine therapy of HCC. For instance, AFP-pulsed DC can stimulate specific cytotoxic T-lymphocytes (CTL) towards AFP-producing HCC cells [13,14]. However, DC-based vaccination protocols failed to induce clinical responses in patients to date, even if tumor-specific CTL were detected [15,16,17].

The presence of a strong immunosuppressive tumor environment characterized by recruitment of regulatory T-cells (Treg) and myeloid-derived suppressor cells (MDSC), the inhibition of T-cell recognition and reduction of Th1-cytokines, seems to be an important reason for the lack of effectiveness of DC-based immunotherapy [18,19,20,21].

CD40L is a potent co-stimulatory molecule expressed on activated CD4^+^-T-helper cells, which can interact with CD40 on DC inducing helper-dependent CTL and enhance the production of Th1 cytokines by DC [22,23,24].

In the present study, intratumoral co-stimulation with CD40L by CD40L-expressing DC (Ad-CD40L-DC) was used for the first time to improve vaccination with murine adenoviral AFP transduced DC (Ad-mAFP-DC) in pre-established subcutaneous (s.c.) and orthotopic HCC in vivo. The impact of this combined strategy on the tumor environment was studied at different time points during the immune response.

## 2. Materials and Methods

### 2.1. Mice and Cell Lines

Eight to twelve week old C3H/HeN mice (Charles River, Sulzfeld, Germany) were maintained under standard SPF conditions. Animal studies were performed according to the local regulatory guidelines. Human embryonic retinoblasts 911 cells [25] were used to propagate E1-deleted adenoviral vectors (Ad). Murine hepatoma cells Hepatoma129 (Hepa129) were obtained by the DCT Tumor Repository, Frederick, Maryland. Hepa129 and murine Hepa1–6 cells (ATCC CRL-1830) were maintained in RPMI1640 with fetal calf serum (PAA, Cölbe, Germany).

### 2.2. Plasmids and Adenoviral Vectors

**Figure 1:**
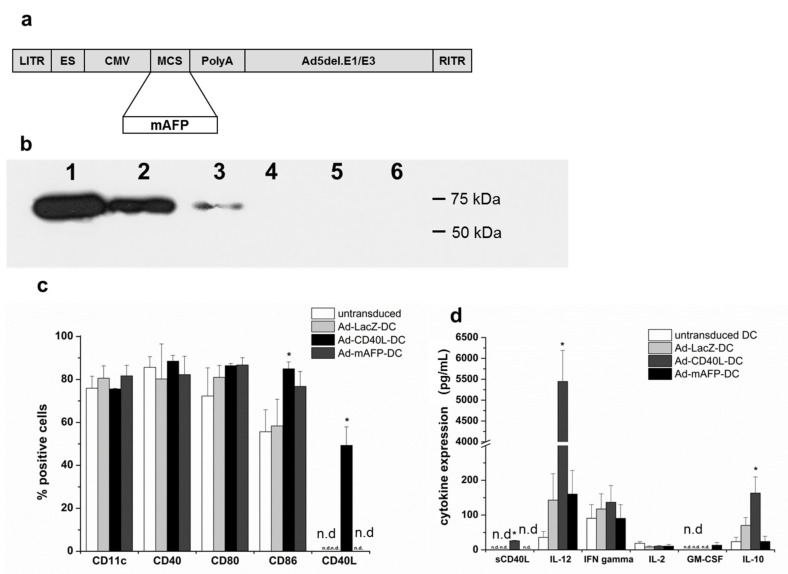
Characterization of Ad-mAFP and Ad-CD40L transduced DC. (**a**) Map of Ad-mAFP. LITR: left inverted terminal repeat; ES: encapsidation signal; CMV: cytomegalovirus immediate early promoter; MCS: multiple cloning site; PolyA: polyadenylation signal; Ad5del.E1/E3: human adenovirus type 5 sequence with deletion of E1-/E3-genes; RITR: right inverted terminal repeat. (**b**) Western Blot detecting mAFP- specific band 1: mAFP-positive Hepa1–6, 2: Ad-mAFP-transduced mDC (MOI 500), 3: Ad-mAFP-transduced mDC (MOI 100), 4: non-transduced mDC, 5: Ad-GFP-transduced mDC (MOI 500), 6: control adenovirus-transduced DC (MOI 500). Samples have equal amounts of protein. (**c**) Flow cytometry of maturation markers of DC 48 h after adenoviral transduction. The percentage of positive cells of the pre-gated CD11c-positive population was analyzed (*n* = 4, * = *p* < 0.05 compared to Ad-LacZ-DC), n.d. = non detectable. Results represent means ± SD of three different experiments. (**d**) Cytokine amounts in the supernatant of DC 48 h after adenoviral transduction. n.d. = non detectable. Results represent means ± SD of three different experiments. (* *p* < 0.05 compared to Ad-LacZ-DC). Significance was determined using the Mann-Whitney-test.

Plasmid BMG-neo-mAFP containing the cDNA of mAFP has been described previously [26]. The cDNA of mAFP was cloned into pShuttleCMV recombined with pAdEasy-1 and transfected into 911 cells to generate mAFP-encoding E1-deleted Ad (Ad-mAFP) as described previously (Stratagene, La Jolla, USA), [27] (Figure 1a). Ad-CD40L (encoding murine CD40L) and Ad-LacZ (encoding E.coli β-galactosidase gene) were kindly provided by Dr. Jesús Prieto, Navarra University, Spain [28].

### 2.3. Stable Transfection of Hepa129-Cells

For stable transfection, lipofectamine (Invitrogen, Karlsruhe, Germany) was used according to the manufacturer’s instructions. Briefly, Hepa129-cells were overlaid with a mixture of 20 µg of BMG-neo-mAFP and lipofectamine. After four weeks of neomycin selection, cells were screened for mAFP-expression. 

### 2.4. Western Blot of mAFP

Amounts of mAFP on DC after adenoviral transduction with Ad-mAFP were verified by Western blot. A rabbit anti-mAFP antiserum (ICN, Costa Mesa, CA) and a horse peroxidase-conjugated secondary anti-rabbit antibody (Santa Cruz, Heidelberg, Germany) were used as previously described [12]. Visualization was achieved by chemiluminescence (Perbio-Science, Bonn, Germany).

### 2.5. Bone Marrow Derived DC and Adenoviral Transduction

DC were generated from bone marrow of mice and cultured with GM-CSF and IL-4 as described previously [29]. Six days after their generation, DC were transduced with adenoviruses (Ad-m CD40L, Ad-mAFP or Ad-LacZ) in RPMI1640 with 2%FCS for 2 h at 37 °C.

### 2.6. Tumor Induction

**Figure 2:**
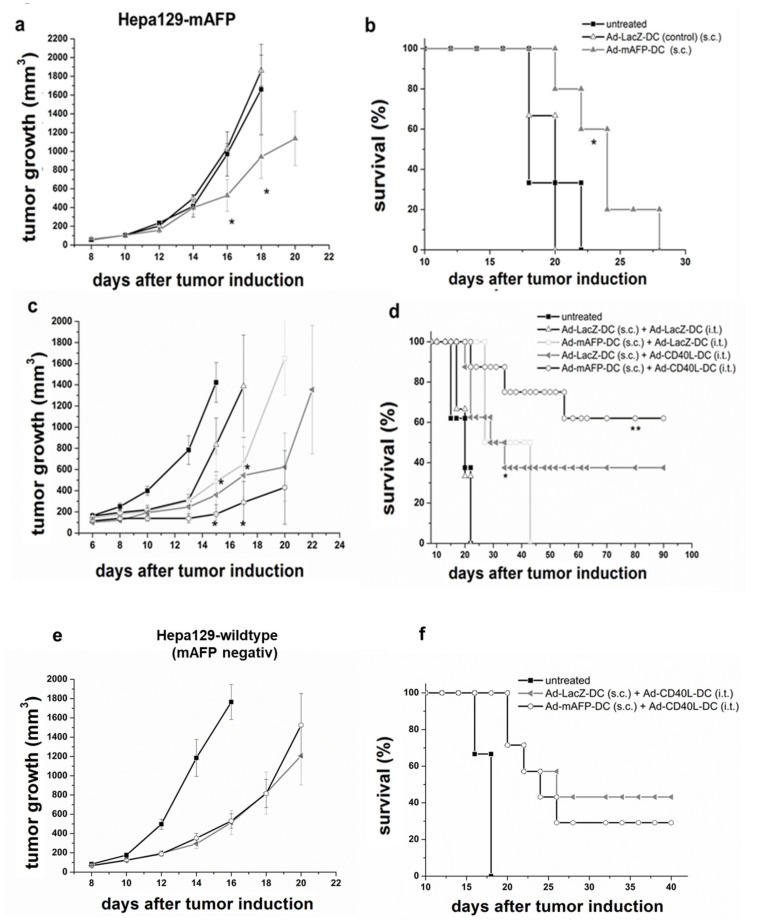
Antitumoral effects of vaccination with Ad-mAFP-DC alone or in combination with Ad-CD40L-DC in pre-established HCC. (**a**,**b**) Tumors were induced s.c. with 10^6^ Hepa129-mAFP-cells. When tumors reached 60.6 ± 16mm^3^ (approximately 7–10 days after tumor induction), 10^6^ Ad-mAFP-DC or 10^6^ Ad-LacZ-DC were injected into the left flank of mice. Graphic shows tumor growth (**a**) and Kaplan-Maier survival curves (**b**) of a representative experiment (*n* = 6 each group) from two different experiments. Data are given as mean tumor volumes (mm^3^) with SEM. (* *p* < 0.05 compared to s.c. Ad-LacZ-DC). (**c**,**d**) Animals were treated s.c. with 10^6^ Ad-mAFP-DC, as described above, followed by an i.t.-injection of 10^6^ Ad-CD40L-DC five days later. Graphics show tumor-growth (**c**) and Kaplan-Maier survival curves (**d**) of one representative experiment (*n* = 8 each group) from three independent experiments. Data are given as mean tumor volumes (mm^3^) with SEM. (* *p* < 0.05, ** *p* < 0.01 compared to s.c.-Ad-LacZ-DC/i.t.-Ad-LacZ-DC). (**e**,**f**) Combined heterotopic DC-treatment in s.c. pre-established AFP-negative HCC (**e**) and Kaplan-Maier survival curves (**f**). Animals were treated as described above. Graphic shows one representative experiment (*n* = 6 each group) from two independent experiments. Data are given as mean tumor volumes (mm^3^) with SEM. The lines marked with circles, triangles or squares were chosen to distinguish different treatments and represent time-points. Significance was determined using the log-rank test.

**Figure 3:**
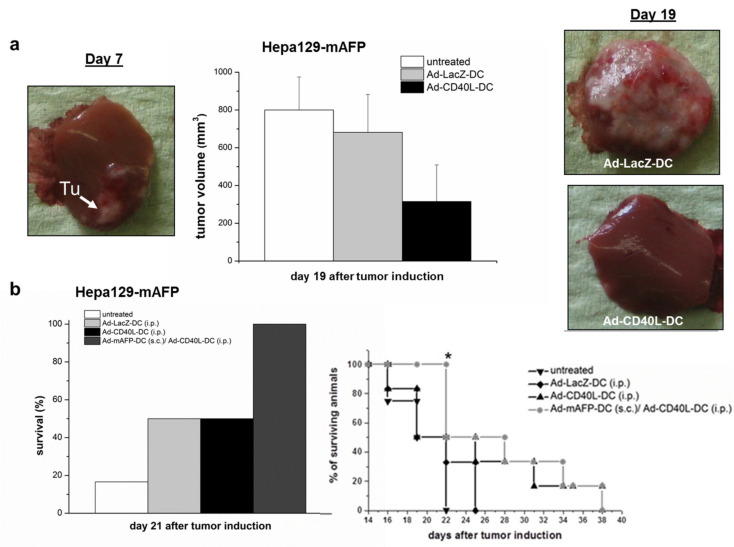
Antitumor effects in pre-established orthotopic HCC. (**a**) Tumor volume of orthotopic HCC after i.p.-treatment with 10^6^ Ad-CD40L-DC 19 days after tumor induction (middle). Data are presented as mean tumor volume ± SD, (*n* = 6 each group). Left: representative tumor at day 7 after tumor induction (before treatment). Right: representative tumors 19 days after tumor induction (12 days after DC-treatment). (**b**) Left: Survival rates of mice bearing orthotopic tumors after treatment with heterotopic DC-injection at day 21 (*n* = 6 each group) Right. The lines marked with circles, triangles or squares were chosen to distinguish different treatments and represent time-points. Kaplan-Maier survival curve. Significance was determined using the log-rank test.

S.c. and orthotopic tumors were induced as described previously [27]. Briefly, 10^6^ Hepa129-mAFP or Hepa129-cells were inoculated s.c. into the right flank of mice. Tumors were monitored by measuring two perpendicular diameters with calipers. For orthotopic tumors, 10^5^ Hepa129-mAFP-cells were injected into the liver after laparotomy (Figure 2a–f and Figure 3a–b).

### 2.7. DC Treatment Experiments

Heterotopic DC treatments were performed. Therefore, mice with s.c. pre-established tumors respectively (approximately six to ten days after tumor induction), were first vaccinated s.c. with 10^6^ Ad-mAFP-DC or Ad-LacZ-DC followed by an intratumoral (i.t.) injection with 10^6^ Ad-CD40L-DC or Ad-LacZ-DC five days later. 

### 2.8. ELISA

**Figure 4:**
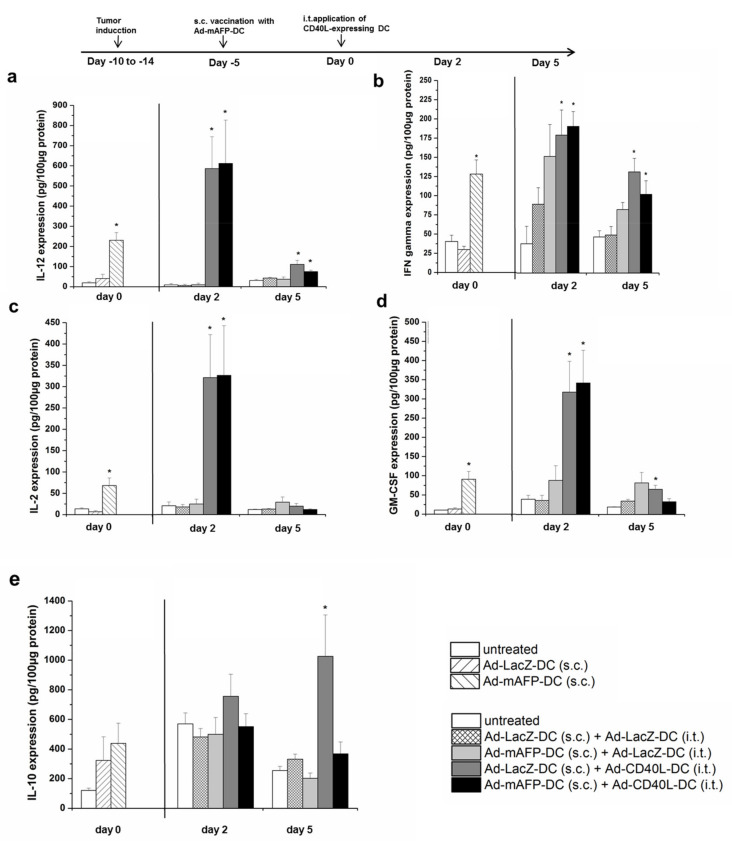
Intratumoral cytokine amount. (**a**–**e**) Mice were treated with the heterotopic combination of a s.c. vaccination with 10^6^ Ad-mAFP-DC or Ad-LacZ-DC followed by an i.t. injection of 10^6^ Ad-CD40L-DC or Ad-LacZ-DC. Analysis of cytokine amount within the tumors was performed on day 0 (after the s.c. vaccination before i.t. injection of Ad-CD40L-DC), on day 2 and 5 after the i.t. injection of Ad-CD40L-DC. Amounts of IL-12, IFN-γ, IL-2, GM-CSF and IL-10 in tumors were measured via ELISA Results represent means ± SEM of three independents experiments (*n* = 8). (* *p* < 0.05 compared to s.c.-Ad-LacZ-DC/i.t.-Ad-LacZ-DC group). Significance was determined using the Mann-Whitney-test.

Levels of IL-12, IFNγ, IL-2, GM-CSF, IL-10 and soluble CD40L on DC-supernatant or protein lysates from explanted tumors were measured using commercially available enzyme-linked immunosorbent assay (ELISA) kits (eBioscience, San Diego, CA, USA) according to the manufacturer’s instructions (Figure 4a–e). 

### 2.9. Flow Cytometry

DC were immuno-phenotyped using PE-, APC- or FITC-conjugated monoclonal antibodies for CD11c, MHC-I, CD40, CD80, CD86 and CD40L (eBioscience, Frankfurt, Germany). To block CD40L, an anti-CD40L antibody (10µg/mL) (Pharmingen, Heidelberg, Germany) was added to the cultures. 

**Figure 5:**
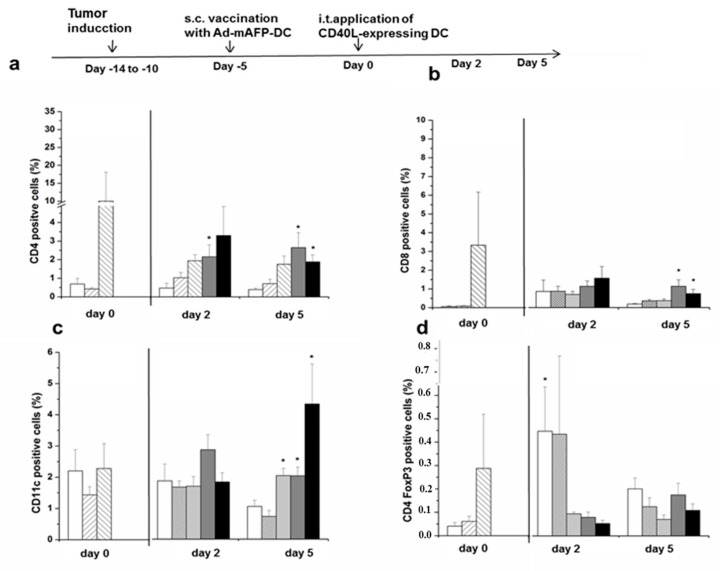

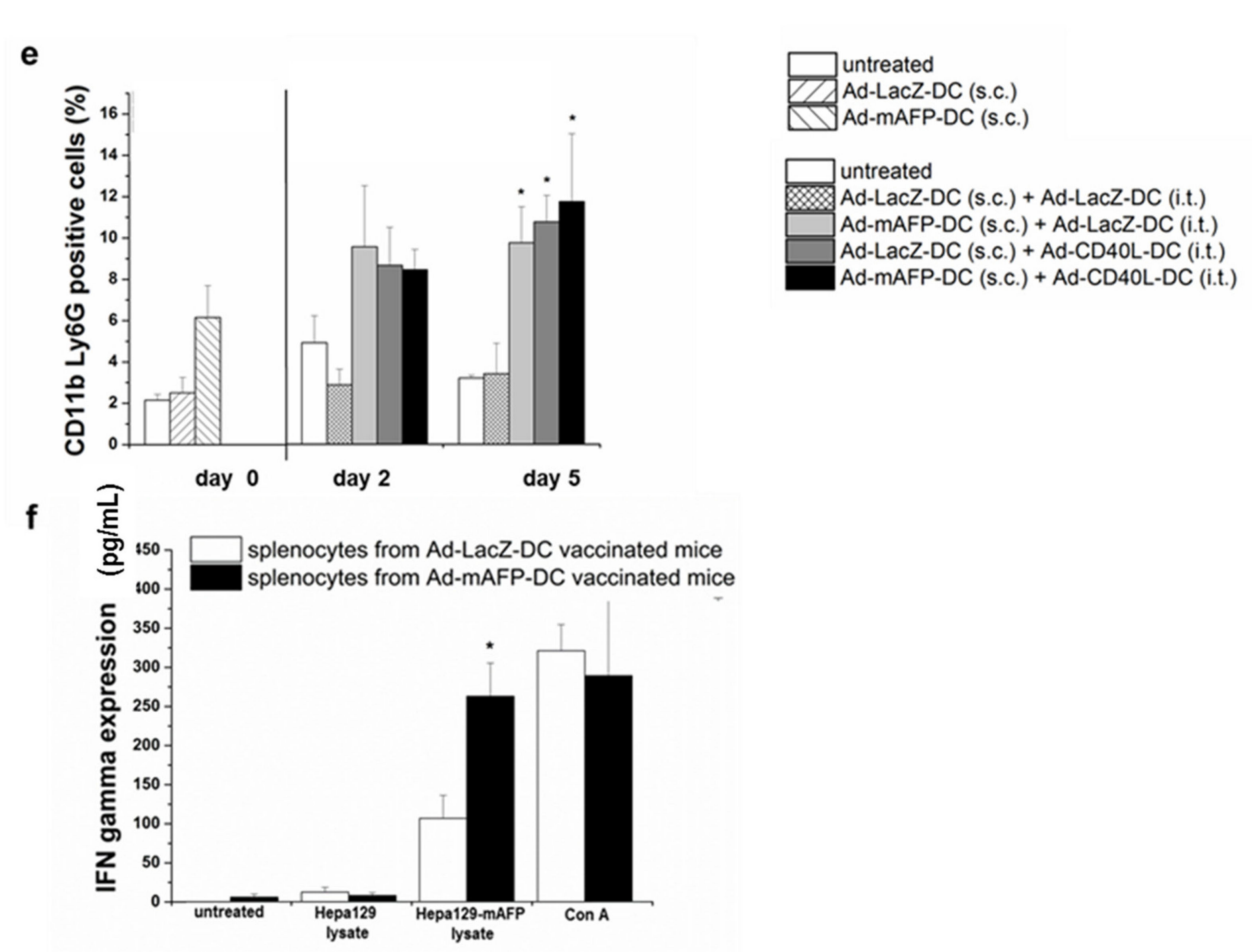
Intratumoral immune cell recruitment. (**a**–**e**) Percentage of CD4^+^-, CD8^+^-T-cells, DC, Treg and MDSC within the total tumor cell suspension was analyzed via flow cytometry. Mice were treated with the heterotopic combination of a s.c. vaccination with 10^6^ Ad-mAFP-DC or Ad-LacZ-DC followed by an i.t. injection of 10^6^ Ad-CD40L-DC or Ad-LacZ-DC. Analysis of immune cell recruitment was performed on day 0 (after the s.c. vaccination before i.t. injection of Ad-CD40L-DC), on day 2 and 5 after the i.t. injection of Ad-CD40L-DC. Results represent means ± SEM of three independents experiments (*n* = 8). (* *p* < 0.05 compared to s.c.-Ad-LacZ-DC/i.t.-Ad-LacZ-DC group). (**f**) Splenocytes were isolated 3 months after s.c. Ad-mAFP-DC or Ad-LacZ-DC vaccination, re-stimulated with tumor cell lysates for 24 h and quantified for IFNγ-secretion by ELISA. Data are given as mean ± SEM. Graphic shows one representative experiment (*n* = 6 each group) from two different experiments. (* *p* < 0.05 compared to s.c.-vaccination with Ad-LacZ-DC splenocytes). Con A: Concanavalin A. Significance was determined using the Mann-Whitney-test or the paired *t*-test (**f**).

Tumors were digested using a collagenase/PBS solution (1:125) for 20 min at 37 °C in a shaker. A single cell suspension was prepared by gentle disruption in a metal 100 μm mesh. Cell suspensions isolated from explanted tumors were first stained with Fc block/rat anti-FcRII-III (from 24G2 hybridoma) in order to bind the CD16 and CD32 receptors located on the surface of monocytes, B lymphocytes, macrophages, granulocytes, NK cells and blood platelets. Afterwards, cells were stained with FITC-, APC-, or PE-conjugated anti-CD3, anti-CD4, anti-CD8 staining as well as anti-CD11c for DC and anti-CD45, anti-CD11b and anti-Gr1 for MDSC (eBioscience) as described previously [29] (Figure 5a–f).

**Figure 6:**
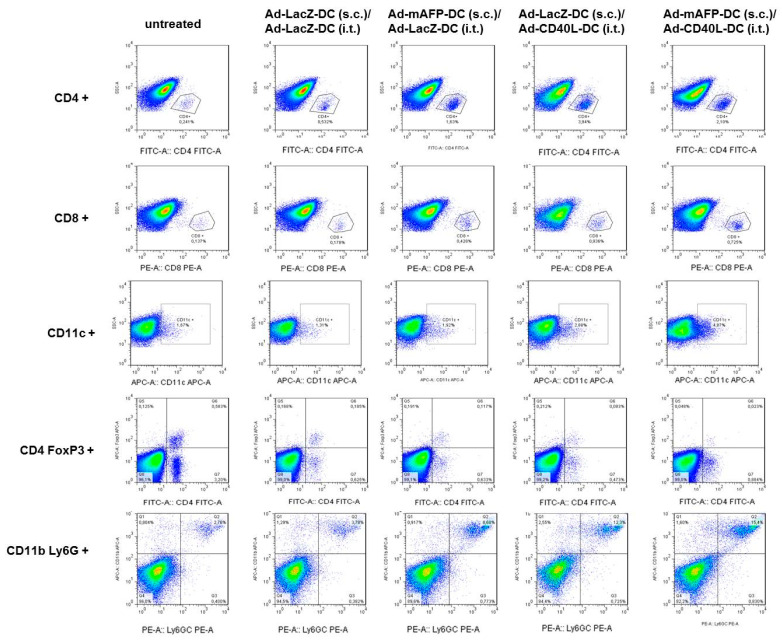
Flow cytometry of CD4, CD8, CD11c, Treg and MDSC of representative tumors.

Treg were detected using the Treg Kit (eBioscience) according to the manufacturer’s instructions. Cell viability was assessed by staining with dye eFluor 506 (eBiosience) or DAPI. Events with very low FSC and SSC were eliminated in order to avoid cell fragments. A viability marker (eFluor 506 or DAPI) was added to remove dead cells from the analysis. Finally, the populations of interest were gated using different antibodies. To gate the T-cells, CD3/CD4 or CD3/CD8-stainings were performed. To gate Treg cells, CD3/CD25/CD4 and FoxP3-stainings were performed. In Figure 6, we show a representative tumor of each combined treatment with the last step of the gating strategy for FACS experiments for different immune cell populations.

Apoptosis was determined by analyzing the subG1-fraction. Briefly, hepatoma cells were harvested and fixed with 70% ethanol, and DNA amounts, which are reduced in apoptotic cells due to degradation/fragmentation, were assessed by propidium iodide staining. Flow cytometry was performed on a FACSCanto II using Diva software and FlowJo7.2.2 (TreeStar Inc., Ashland, OR, USA).

### 2.10. Isolation of Splenocytes and IFNγ-Secretion Assay

Splenocytes of mice were isolated as described previously [29]. Splenocytes were restimulated with 100 µg tumor lysate and the presence of tumor specific effector cells was analyzed through IFNγ-secretion assay. As positive control, a restimulation with ConA (Concanavalin A, a lectin carbohydrate-binding protein) was performed.

### 2.11. Caspase Detection in Tumors

Apoptosis in tumors was analyzed by detection of caspase activity, which was measured by cleavage of specific fluorogenic substrates: Ac-DEVD-7-amino-4-trifluoromethyl coumarine (afc) (Bachem, Heidelberg, Germany) for caspase-3, Ac-LETD-afc (Alexis, Grünberg, Germany) for caspase-8 and Ac-LEHD-afc (Bachem) for caspase-9. After homogenization of tumors, corresponding fluorogenic substrate was added. Fluorescence intensity was measured in a Shimadzu RF-5301PC fluorometer. Caspase activity was calculated from the slope as fluorescence units/mg protein/minute of reaction time and converted to picomoles of substrate cleaved/mg protein/minute based on a standard curve for afc. 

### 2.12. Statistical Analysis

For descriptive statistics, means and standard deviation (SD) or standard errors of the mean (SEM) are given. A paired *t*-test and the Mann-Whitney-test were used to analyze significance. Survival rates are presented as Kaplan-Meier curves, significance was determined using the log-rank test. A *p*-value of <0.05 was considered significant. Data were analyzed by the statistical software GraphPad Instat Version 3.1.

## 3. Results

### 3.1. Adenoviral Transduction of DC with Ad-mAFP and Ad-CD40L

A specific band was detected for mAFP in Ad-mAFP-DC at 69 kDa by Western blot (Figure 1b and Figure S1).

In order to elucidate the effects of Ad-hCD40L transduction on cytokine amount, we analyzed the supernatants of the DC by ELISA. After transduction with Ad-CD40L membrane, CD40L was detected in 50% of DC and the concentration of soluble CD40L was 26+/–1.3 pg/mL in their supernatant (Figure 1c,d). CD40/CD40L-interaction between DC increased significantly the concentration of CD86 on DC (84.9 ± 3.2%) (Figure 1c) as well as the concentration of IL-12- to >5000 pg/mL/10^6^ DC (Figure 1d). Additionally, increased amounts of the Th2 cytokines, such as IL-10 on DC were observed.

### 3.2. Induction of mAFP-Positive Hepatocellular Carcinoma in C3H Mice

For induction of mAFP-positive tumors, syngenic hepatoma Hepa129-cells were stably transfected with BMG-neo-mAFP. Tumor growth of Hepa129-mAFP was similar to wild type Hepa129 tumors and resulted in palpable tumors already six to ten days after s.c.-challenge with 10^6^ Hepa129-mAFP-cells (Figure 2a–f). Animals were sacrificed three weeks later due to advanced tumor growth (1500–2000 mm^3^). Hepa129-mAFP tumors showed poor immunogenicity with down-regulation of MHC-I molecules (10 ± 3.4% of cells) and high concentration of immunosuppressive cytokines, such as TGF-β (924 ± 135 pg/mL), IL-10 (106 ± 14 pg/mL) or VEGF (830 ± 339 pg/mL) in the supernatant. Moreover, Hepa129-mAFP-derived tumors exhibited almost no infiltration with CD4^+^-, CD8^+^-T-cells and DC, but high infiltration with Treg and MDSC, similar to patients with HCC [30].

### 3.3. Antitumor Effects of s.c. Vaccination with Ad-mAFP-DC on Pre-Established HCC

In this experiment we examined whether s.c. vaccination with Ad-mAFP-DC achieved antitumoral effects in pre-established tumors, in both Hepa 129-mAFP. The Kaplan-Maier survival curves shows that Hepa129-mAFP-bearing mice benefit from the s.c. -vaccination with Ad-mAFP-DC. 

As shown in Figure 2a,b, s.c.-vaccination with Ad-mAFP-DC could induce a significant reduction and slight delay of tumor-growth compared to Ad-LacZ-DC, but not a complete remission of the tumor.

### 3.4. Antitumor Effects of s.c. Vaccination with Ad-mAFP-DC in Combination with i.t. Application of Ad-CD40L-DC in Pre-Established HCC

To overcome the poor efficacy of Ad-mAFP-DC towards pre-established HCC, Ad-CD40L- DC were injected i.t. five days after Ad-mAFP-DC-vaccination. As hypothesized, a single i.t.-injection of Ad-CD40L-DC in combination with s.c. Ad-mAFP-DC-vaccination improved significantly the antitumoral immune response compared to Ad-LacZ-DC-vaccination and i.t.-injection of Ad-LacZ-DC (control treatment), reaching complete tumor remission in 62.5% of animals and long-term survival of more than 100 days (shown in Figure 2c,d). In contrast, 0% of pre-established tumors were rejected by s.c.-vaccination of Ad-mAFP-DC or Ad-LacZ-DC combined with i.t.-injection of Ad-LacZ-DC and only 37.5% by s.c.-vaccination of Ad-LacZ-DC (control vaccination) combined with i.t.-injection of Ad-CD40L-DC.

Moreover, the combined immunotherapy seems to be AFP-specific, since vaccination with Ad-AFP-DC combined with i.t.-injection of Ad-CD40L-DC did not differ from a vaccination with Ad-LacZ-DC combined with i.t.-injection of Ad-CD40L-DC in tumor-bearing mice with AFP-negative Hepa129-tumors (Figure 2e,f). At 40 days after tumor induction only 30 and 44% of the tumor-bearing mice were still alive after treatment with s.c.-Ad-mAFP-DC/i.t.-Ad-CD40L-DC or s.c.-Ad-LacZ-DC/i.t.-Ad-CD40L-DC respectively. 

### 3.5. Antitumor Effects in Pre-Established Orthotopic HCC

Combining a s.c.-vaccination with Ad-mAFP-DC with a intraperitoneal (i.p.) instead of i.t. injection of Ad-CD40L-DC was analyzed also on orthotopic HCC. 

DC-treatments were performed seven days after tumor induction, when the tumor was recognizable (Figure 3a, left). To test the antitumoral effects of Ad-CD40L-DC alone, animals were injected with 10^6^ Ad-CD40L-DC i.p. As shown in Figure 3a middle/right, a significant reduction of tumor-growth was documented (315+/−194 mm^3^) compared to Ad-LacZ-DC (682 ± 200 mm^3^) (*p* = 0.009) at day 19. Furthermore, at this time-point, detectable tumor-burden and malignant ascites were found only in 50% and 30% of animals treated with Ad-CD40L-DC respectively, whereas 100% of control animals already showed considerable tumor-mass and malignant ascites.

Combining heterotopic s.c.-inoculation with 10^6^ Ad-mAFP-DC followed by i.p.-injection of 10^6^ Ad-CD40L-DC elicited increased survival compared to control treatments with Ad-LacZ-DC (i.p.) or Ad-CD40L-DC (i.p.). Only the combined heterotopic s.c.-inoculation with Ad-mAFP-DC followed by i.p.-injection of Ad-CD40L-DC elicited significant increased survival compared to control treatments with Ad-LacZ-DC or Ad-CD40L i.p. on day 21 (Figure 3b). 

### 3.6. Changes in the Intratumoral Cytokine Amounts

In a further experiment, tumor-bearing animals were treated with the above described different combination therapies. Analysis of cytokine levels within the tumors was performed after the vaccination with Ad-LacZ-DC or Ad-mAFP-DC (day 0). Further mice were sacrificed 2 and 5 days after the additional i.t. treatment with Ad-CD40L-DC or Ad-LacZ-DC (control) in order to determine the changes on cytokine levels due to the additional intratumoral treatments. 

Interestingly, tumors of mice vaccinated with Ad-mAFP-DC (6–10 days after tumor induction) already showed changes within the tumor into a shift towards a Th1 cytokine milieu, expressing significantly more IL-12, IFNγ, IL-2 and GM-CSF compared to mice vaccinated with Ad-LacZ-DC (*p* < 0.05), (Figure 4a–d). Two days after the i.t.-injection with Ad-CD40L-DC and independent of the performed vaccination, a further strong up-regulation of Th1 cytokines was observed within the tumors compared to the controls (s.c.-vaccination Ad-LacZ-DC/i.t.-injection Ad-LacZ-DC) (*p* < 0.005). Moreover, five days later, the amount of Th1-cytokines, such as IL-12 or IFN-gamma within the tumor was still elevated, indicating long-term Th1 milieu within the tumors due to the additional i.t. CD40L co-stimulation. In contrast, in Ad-mAFP-DC-vaccinated mice which were subsequently i.t. treated with control DC (Ad-LacZ-DC), initiated Th1 cytokines were disappeared. Furthermore, only tumors treated with s.c. Ad-mAFP-DC/i.t. Ad-CD40L-DC showed reduced IL-10-amount, but not tumors with s.c. AdLacZ-DC/i.t.-Ad-CD40L-DC, (Figure 4e).

### 3.7. Recruitment of Immune Cell Populations in the Tumors

In the same above described experiment, we now analyzed the effects of Ad-mAFP-DC or Ad-LacZ-DC vaccinations (day 0) and of the different combined treatments with i.t. Ad-CD40L-DC or Ad-LacZ-DC (control treatment) on the infiltration of different immune populations, such as CD4+ and CD8+ T-cells, DC, Treg and MDSC within the tumors using flow cytometry.

As shown in Figure 5a,b and Figure 6 corresponding to the Th1 cytokine shift, already a s.c.-inoculation with Ad-mAFP-DC resulted in an increased infiltration of CD4^+^- and CD8^+^-T-cells in the tumors compared to Ad-LacZ-DC-vaccination. However, an increase of Treg was also detected after vaccination with Ad-mAFP-DC. Two and five days after the i.t. injection with Ad-CD40L-DC, a further recruitment of CD4^+^- and CD8^+^-lymphocytes was observed independent of the vaccination performed. Furthermore, a higher recruitment of DC was found in the group of animals treated with the combination of s.c. Ad-mAFP-DC/i.t. Ad-CD40L-DC (Figure 5c). In order to differentiate the CD4^+^-T-helper cell population from Treg, we further analyzed the CD25- and Foxp3-amounts. As shown in Figure 5d, proportions of Treg were slightly lower in the combination group of s.c. Ad-mAFP-DC/i.t. Ad-CD40L-DC than in the all the other groups. Interestingly, the MDSC population was also significantly increased in the groups with s.c.-Ad-mAFP-DC and with it-Ad-CD40L-DC compared to the control group (s.c.-Ad-LacZ-DC/i.t.-Ad-LacZ-DC) (*p* < 0.05), (Figure 5e).

### 3.8. Induction of Tumor-Specific Effector Cells

The presence of tumor-specific effector cells was analyzed in spleens from mice after s.c. vaccination with Ad-mAFP-transduced DC and compared with a control vaccination with Ad-LacZ-DC. As expected, splenocytes derived from Ad-mAFP-DC-vaccinated mice produced significantly more IFNγ than splenocytes from Ad-LacZ-DC-vaccinated mice after re-stimulation with Hepa129-mAFP lysate but not after re-stimulation with Hepa129 lysate (*p* = 0.0202), (Figure 5f).

### 3.9. Induction of Tumor Cell Apoptosis by Ad-CD40L-DC

To assess CD40L-induced apoptosis as a further antitumoral mechanism of i.t. Ad-CD40L-DC administration, two experiments were performed. To determine the apoptosis rate in vitro, Hepa129-mAFP and Hepa1–6 cells were cultivated with supernatant from CD40L-expressing DC and the SubG1 fraction in these cells was then measured by flow cytometry. In vivo, the enzymatic detection of caspase 3 activity in protein lysate of HCC treated with i.t. Ad-CD40L-DC was carried out by flow cytometry as described in the methods section.

**Figure 7:**
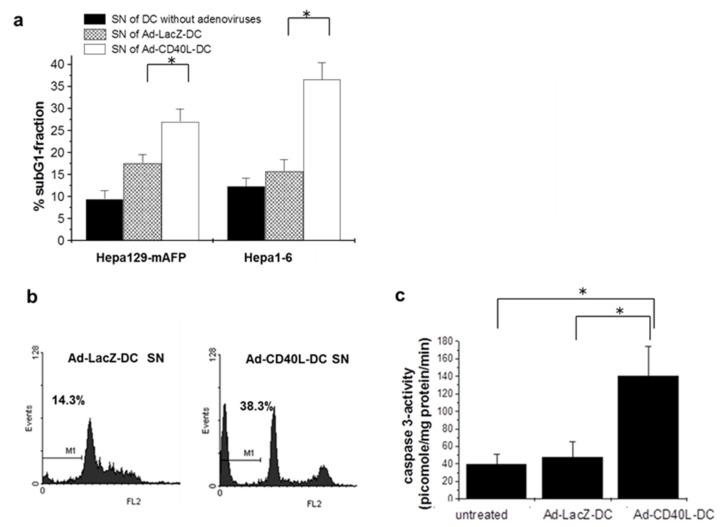
Analysis of tumor cell apoptosis. (**a**) Apoptosis assessment by detection of the sub-G1-fraction in tumor cells 72 h after culturing with the supernatant (SN) from Ad-CD40L-DC. Data are shown as mean ± SD from one representative experiment of four independent experiments. (* *p* < 0.05). (**b**) Flow cytometry of two representative samples of Hepa1–6 cells subG1-fraction after culturing with the SN of Ad-CD40L-DC or Ad-LacZ-DC. (**c**) Caspase-3 activity in protein lysates from explanted tumors after i.t.-injection with Ad-CD40L-DC. Mean ± SD of activity (AFC= turnover in pmol/mg protein/min) is shown (*n* = 4 each group). (* *p* < 0.05). Significance was determined using Mann-Whitney-test (**a,b**) or the paired *t*-test (**c**). Con A: Concavalin A.

Interestingly, we detected significant increase of subG1-fraction in Hepa129-mAFP- and Hepa1–6-cells (Figure 7a) after culturing with the supernatants from Ad-CD40L-DC (*p* < 0.01), (Figure 7b,c), indicating induction of tumor cell apoptosis by Ad-CD40L-DC. In vivo, significantly higher caspase-3 activity was found in all tumors treated i.t. with Ad-CD40L-DC compared to controls, (*p* = 0.0013 and *p* = 0.03, respectively), (Figure 7). For caspase-8 and -9, no differences were detected (not shown).

## 4. Discussion

In first clinical trials, vaccinations against tumor-associated antigens (TAA), such as Alpha-Fetoprotein (AFP) using dendritic cells (DC) failed to achieve effective immune responses towards hepatocellular carcinoma (HCC) [13,15,16]. Apoptosis resistance, lack of co-stimulation and a strong immunosuppressive tumor environment have been described as mechanisms for the tumor immune escape [18,19]. In this study, i.t. co-stimulation with CD40L by using adenoviral transduced DC with CD40L-coding adenoviruses could synergistically improve the antitumoral effects of vaccination with DC transduced with murine AFP-coding adenoviruses in pre-established HCC in vivo. Combined strategy causes rapid and profound changes in the tumor environment with enhanced Th1-cytokine amount, strong tumor infiltration of CTL and DC, and higher tumor apoptosis, leading to effective tumor regression of HCC. Thus, combination of s.c. vaccination with Ad-mAFP-transduced DC with i.t. Ad-CD40L-DC represents a promising tool for improving tumor-antigen DC-based immunotherapy of HCC.

DC as professional antigen presenting cells possess a high potential for cancer immunotherapy, as they are able to initiate T-cell-mediated immune response against TAA. In this context, cellular immunotherapy with autologous DC pulsed with specific TAA (sipuleucel-T) prolonged median survival of patients with advanced prostate cancer and was approved in the USA, opening a new period in DC-based cancer immunotherapy [31,32,33]. 

AFP is a well-characterized TAA, expressed in more than 50% of patients with HCC. In the past, we and others demonstrated the presence of AFP-specific T-cells in patients with HCC. Furthermore, DC pulsed with AFP can stimulate specific CTL towards AFP-producing HCC cells, suggesting that AFP may be an attractive target for DC-based immunotherapy of HCC [12,14]. However, initial clinical trials targeting AFP failed to achieve any clinical responses despite the induction of AFP-specific CD8^+^-T-cells [15]. 

In order to overcome the difficulties of TAA-pulsed DC as vaccine, we returned in this study to the pre-clinical situation using a poor immunogenic AFP-expressing HCC model using mAFP stably transfected murine hepatoma Hepa129-cells. After challenging with Hepa129-mAFP-cells, tumor growth of HCC was very aggressive and showed increased recruitment of Treg, down-regulation of MHC-molecules, reduced amount of Th1 cytokines and poor infiltration with CD4^+^- and CD8^+^-T-cells or mature DC similar to human HCC [21].

For TAA pulsing, DC were transduced with an Ad encoding for mAFP. Due to the amount of the whole protein in the DC all potential mAFP-epitopes can be presented in the context of MHC-I and –II molecules without need of peptide identification. Moreover, adenoviral transduction enhanced MHC-amount, co-stimulatory molecules on DC and IL-12-concentration, suggesting protection from the known suppressive effect of AFP on DC-maturation [34]. In our in vivo experiments, the control treatment with s.c. Ad-LacZ-DC/i.t.-Ad-LacZ-DC was able to induce a low immune response towards HCC. However, this response was not clinically significant compared to untreated animals. Dendritic cells can be activated due to adenoviral virus transduction due to the activation of the Toll-like receptor 9 (TLR9) [35]. When we used Ad-CD40L, DC expressed highly amounts of Th1 cytokine, such as IL-12, but also Th2 cytokines, such as IL-10. AdmCD40L-transduced DC express both forms of CD40L, as membrane-bound (m)CD40L and as soluble (s)CD40L. In a previous work, we show that the effect of sCD40L seemed to induce mainly Th2 cytokines/chemokines, whereas an endogenous expression of mCD40L induced a clear Th1 shift on the supernatant of DC as observed in our transwell experiments and by blocking (s)CD40L [24].

As shown in the results, i.t. Ad-CD40L-DC independent of the vaccination showed a significant tumor-reduction and significant prolonged survival compared to mice treated with i.t.-Ad-LacZ-DC (controls). These results are in line with our previous work using CD40L-expressing DC as monotherapy towards subcutaneous HCC [25]. In the current report, we show that a s.c.-vaccination with Ad-mAFP-DC effectively synergizes with i.t.-injection with Ad-CD40L-DC, thus achieving complete tumor remission in 62% of tumor-bearing animals. This novel combined heterotopic immunotherapy also improved antitumoral effects towards pre-established orthotopic tumors. In AFP-negative Hepa129-tumors a clear difference in tumor size and survival for both i.t.-Ad-CD40L-DC-treated mice independent of vaccination compared to untreated mice was also observed. These results confirm also in AFP-negative tumors the potential of i.t.-application of Ad-CD40L-DC by activating DC and CTLs due to other tumor antigens. However, the immune response was not enhanced by s.c.-vaccination with Ad-mAFP-DC vs. Ad-LacZ-DC in AFP-negative tumors, indicating that the immune response was AFP-specific.

Since several i.t. therapeutic approaches are well-established for the treatment of HCC, i.t.-injection of Ad-CD40L-DC may also be feasible for patients with HCC in combination with s.c. Ad-mAFP-DC. Moreover, i.t.-application of Ad-CD40L-DC would not induce any significant side effects compared to the systemic administration of CD40L [36,37,38]. 

In order to dissect the immunological effects after DC-treatments in the tumor environment, we analyzed the cytokine amount and the recruitment of different immunological cell populations at different time points during the immune response. After the s.c.-vaccination with Ad-mAFP-DC alone, we indirectly could detect AFP-specific T-cells, as shown in IFNγ-secretion assays from splenocytes from vaccinated mice compared to controls. Induction of AFP-specific T-cells seems to initiate an early increase of Th1 cytokines, such as IL-12, IFNγ, GM-CSF and IL-2, as well as enhanced recruitment of CD4^+^- and CD8^+^-T-cells within these tumors compared to control-vaccinated animals (s.c. Ad-LacZ-DC). However, the initiated changes within the tumors were only transient and insufficient to mount an effective immune response, clearly explaining the failed antitumoral effect: two and five days after the i.t. injection with Ad-LacZ-DC, animals vaccinated with Ad-mAFP-DC showed again a reduction of Th1 cytokines and consequently reduced infiltration with effectors cells. These results are in line with the data from the clinical trials using DC as vaccine in patients with HCC, which also failed to achieve clinical responses, even if tumor-specific effector cells were induced. By contrast, i.t.-injection with Ad-CD40L-DC/vaccinated with Ad-mAFP-DC not only maintained but also strongly expanded the amount of Th1 cytokines, leading to a strong infiltration with CD4^+^-, CD8^+^-T-cells and mature DC. This early and maintained shift to a Th1 milieu accompanied by a strong infiltration of lymphocytes seems to be crucial to overcome the immune tolerance and to induce effective remission of pre-established tumors and represents a key knowledge for understanding the effectiveness of the presented approach. 

Interestingly, the combination therapy with s.c. Ad-mAFP-DC and i.t. Ad-CD40L-transduced DC could reverse the production of IL-10 within the tumors probably induced by soluble CD40L. However, the cellular interaction between CD40 and CD40L seems to be relevant for the Th1-shift. Due to the s.c.-vaccination with Ad-mAFP-DC, we expect an effective priming of AFP-specific T-cells explaining more effective tumor infiltration with CD4+-, CD8+-T-cells, more release of tumor antigens and more infiltration of DC within the tumors, as shown in Figure 5c (day 5). Due to the infiltration of DC, we expect more cellular CD40/CD40L-interaction, reducing the sCD40L effect on CD40. This would explain the reduction on IL-10 amounts within the tumor tissue.

Treg (CD4^+^/CD25^+^/Foxp3^+^) are immunosuppressive cells in the tumor microenvironment and correlates with tumor progression also in HCC [39]. In a previous study, we demonstrated that Treg cells contributed to the suppression of anti-tumor immunity in HCC via several pathways. One of these signal pathways included the interaction between Treg cells and effector cells due to checkpoint inhibition (PD-L1/PD-1). Furthermore, Granito et al., showed that Treg cells are involved in immune-related adverts events due to the novel immune checkpoint inhibitors [40,41].

In our experiment, after a vaccination with Ad-mAFP-DC an increase not only of CD4+- and CD8+-T-cells, but also of Treg, was detected. Both effector T cells (CD4+ and CD8+ T-cells) and Tregs appear to be initially primed by the vaccination with Ad-mAFP-transduced DC, which could explain the insufficient effect of a s.c. vaccination with Ad-mAFP-transduced DC towards established HCC. Interestingly and in line with recent published works, i.t. Ad-CD40L-DC appears to reduce amounts of Treg and IL-10 in the tumors, contributing to the enhanced immune response [42,43,44]. 

MDSC comprise a mixture of monocytes/macrophages, granulocytes, and DC at different stages of differentiation. In patients with HCC, MDSC have also been shown to be associated with impaired T-cell functionality and to induce Foxp3 and IL-10-in CD4^+^-T-cells as a further mechanism of immunosuppression [30]. However, we observed in our model an increase of MDSCs in all treated groups with s.c. Ad-mAFP-DC, i.t. Ad-CD40L-DC or their combination. In all these groups, we also observed high levels of IFN-gamma within the tumors (Figure 4b). As shown by Pan et al. the development of Tregs mediated by MDSCs required antigen-associated activation of tumor-specific T-cells and depended on CD40 and IFN-γ signaling by MDSCs and could explain increased of MDSCs in our model [45].

The effects of CD40 signaling have been described to be multifaceted [46]. In our study, we observed that tumors treated locally with Ad-CD40L-DC showed higher release of caspase 3. Hence, apoptosis induction appears to be a further mechanism of tumor-inhibition of i.t. Ad-CD40L-DC in our model [47,48]. It is possibly related to increased IL-12 and IFNγ levels within the tumors [49]. In vitro, we showed apoptosis of tumor cells after culturing with the supernatant from Ad-CD40L-DC. As murine hepatoma cells Hepa129-mAFP and Hepa1–6 express small amounts of CD40 (about 10–30% of cells, not shown), apoptosis may also be mediated by direct CD40/CD40L interaction between soluble CD40L secreted in the supernatant of Ad-CD40L-DC and CD40 from tumor cells.

Thus, the presented approach seems to effectively combine different immunological and pro-apoptotic mechanisms which synergistically converge into the tumor, clearly improving the antitumoral effects of a conventional vaccination protocol with Ad-mAFP-DC. To our knowledge, co-stimulation with CD40L in order to improve a vaccination with AFP-pulsed DC for immunotherapy of HCC have never been described before. However, our study has several limitations. Transferring this protocol to patients in clinical practice may not be easy. The use of adenoviral transduced DC requires an expensive and complex cell culture process, which may limit their availability for clinical use. Furthermore, our results are limited by the use of an implanted murine HCC model. The effects of the underlying etiology of HCC, including a fibrotic/cirrhotic liver, cannot be analyzed in this model. Finally, the different effects of soluble or membrane CD40L may also increase immunosuppressive cell populations or cytokines, such as MDSC or IL-10 within the tumors and should be further explored.

## 5. Conclusions

In conclusion, s.c. inoculation with Ad-mAFP-DC combined with i.t. injection of DC engineered to express CD40L causes rapid and profound changes in the tumor environment with enhanced Th1-cytokine amount, strong tumor infiltration of CTL and DC, and higher tumor apoptosis, leading to effective tumor regression of HCC. Thus, CD40L co-stimulation represents a promising tool for improving tumor-antigen DC-based immunotherapy of HCC, which should be tested in clinical trials.

## Data Availability

The data presented in this study are available on request from the corresponding author. The data are not publicly available due to private restrictions.

## Supplementary Materials

The following are available online at https://www.mdpi.com/article/10.3390/cancers13133375/s1, Figure S1: Western blots with different exposure times.
