# Supplementary material for: Alpha-Fetoprotein- and CD40Ligand-Expressing Dendritic Cells for Immunotherapy of Hepatocellular Carcinoma
Source: Cancers (Basel). 2021 Jul 5;13(13):3375. doi: 10.3390/cancers13133375 (PMC8269346; doi:10.3390/cancers13133375)
Supplement: Supplementary file 1 [file cancers-13-03375-s001.zip › cancers-1238985-supplementary.pdf]

Supplementary Materials

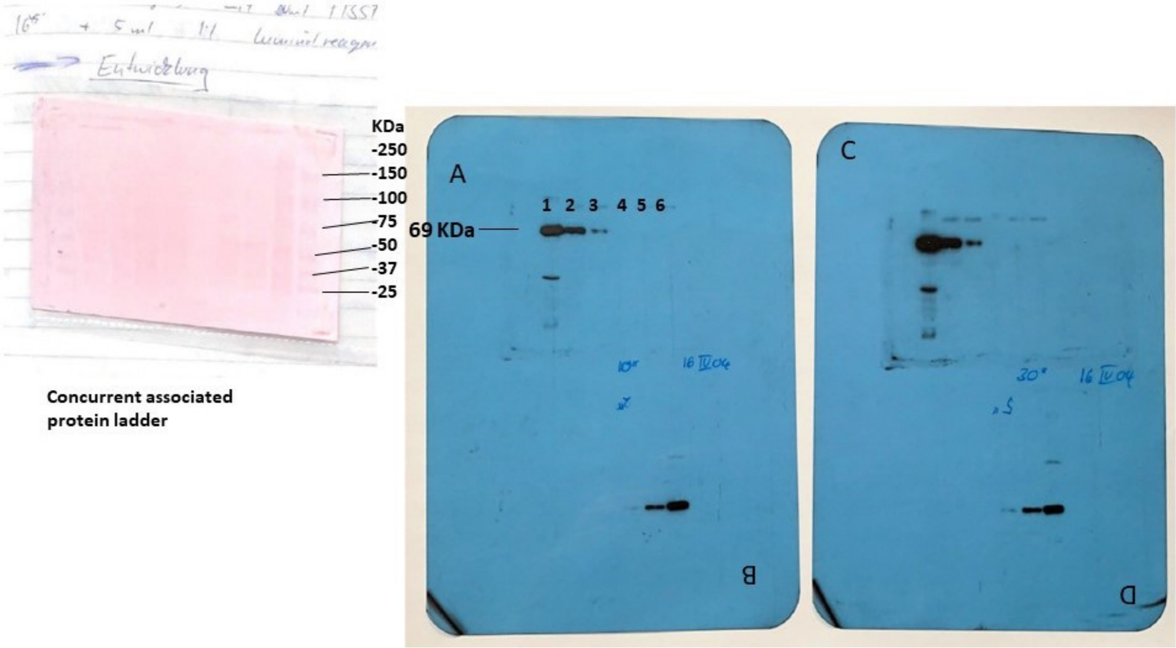

Figure S1. Western blots with different exposure times (**A–D**): 2″, 5″, 10″, 30″. For the manuscript we used the Western blot with 10 "exposure time (=A), the band at 69 KDa represents murine Alpha-Fetoprotein
